# A survey of the Queensland healthcare workforce: attitudes towards dementia care and training

**DOI:** 10.1186/1471-2318-13-101

**Published:** 2013-09-30

**Authors:** Catherine M Travers, Elizabeth Beattie, Melinda Martin-Khan, Elaine Fielding

**Affiliations:** 1 Queensland Dementia Training Study Centre, Queensland University of Technology (QUT), Level 6, N Block, Victoria Park Rd., Kelvin Grove Qld 4059, Australia; 2 School of Nursing, Dementia Collaborative Research Centre: Carers and Consumers, QUT, Level 6, N Block, Victoria Park Rd., Kelvin Grove Qld 4059, Australia; 3 Centre for Research in Geriatric Medicine, The University of Queensland, Princess Alexandra Hospital, Ipswich Rd., Woolloongabba Qld 4102, Australia

**Keywords:** Dementia, Attitudes of health professionals, Education, Training, Nursing, Allied health

## Abstract

**Background:**

Positive attitudes of healthcare staff towards people with dementia promote higher quality care, although little is known about important factors that underlie positive attitudes. Key aims of this project were to explore the relationships between staff attitudes towards dementia, self-confidence in caring for people with dementia, experience and dementia education and training.

**Methods:**

A brief online survey was developed and widely distributed to registered nurses and allied health professionals working in Queensland in 2012. Regression analyses were performed to identify important predictors of self-confidence in caring for people with dementia and positive attitudes towards people with dementia.

**Results:**

Five hundred and twenty-four surveys were completed by respondents working in a range of care settings across Queensland. Respondents were predominantly female (94.1%), and most were registered nurses (60%), aged between 41 and 60 years (65.6%). Around 40% regularly worked with people with dementia and high levels of self-confidence in caring for this population and positive attitudes towards people with dementia were reported. The majority of respondents (67%) had participated in a dementia education/training activity in the past 12 months. More experience working with people with dementia predicted greater self-confidence while recent participation in a dementia education/training and higher self-confidence in caring for a person with dementia significantly predicted more positive attitudes towards people with dementia.

**Conclusions:**

These results confirm the importance of self-confidence and dementia education in fostering positive attitudes and care practices towards people with dementia. Our results also indicate that the demand for ongoing dementia education is high amongst health care workers and it is recommended that regular dementia education/ training be provided and promoted for all healthcare personnel who work with people with dementia.

## Background

Dementia is a leading cause of disability and disease burden among older people [1], and the prevalence of dementia in Australia is expected to more than double from an estimated 300,000 in 2011 to nearly 600,000 in 2030–2031, due primarily to demographic ageing [1,2]. People with dementia rely heavily on health and aged care services and the expected increase in the number of people with dementia will require a considerable expansion in the health and aged care workforce if this population is to receive adequate care [1]. Little is known, however, about the preparedness of the Australian healthcare workforce to care for this population and while limited research has been undertaken to date, studies indicate that public and professional knowledge of dementia is generally low and that many nurses have had little or no dementia training [3].

The provision of high quality care for people with dementia is dependent on staff having a high level of dementia care knowledge and positive attitudes towards people with dementia [4-7]. Importantly, it has been shown that staff who have good knowledge of dementia care have more positive attitudes towards people with dementia [8], which in turn, have been shown to be associated with better quality of care and better quality of life in residents with dementia living in long-term care facilities [4-7]. It has also been shown that dementia training programs can improve staff knowledge, attitudes and confidence in caring for patients with dementia [3,9-11].

There are very limited data regarding the attitudes of Australian healthcare professionals towards people with dementia, their levels of self-confidence, experience, and dementia education and training. Hence, this study was undertaken to obtain data in relation to these issues in a large sample of healthcare professionals working in Queensland, Australia. Additional aims were to further explore the associations between attitudes towards people with dementia, self-confidence in caring for people with dementia, experience, training and education. It was hypothesised there would be positive relationships between education, training and self-confidence and positive attitudes towards people with dementia.

## Methods

An online survey, designed to be brief and completed anonymously, was developed. An initial draft of the survey questions was prepared by the Project Coordinator and distributed to members of the advisory group (primarily senior nurses with specialist dementia knowledge) to ensure the survey remained brief yet included all key questions identified in the project aims. The face validity of the survey was tested by distributing it to members of the advisory group and associates (healthcare professionals with experience in dementia care and researchers). Twenty-six surveys were distributed in this manner and comments regarding the survey questions were received from 10 people, following which questions were revised to improve clarity.

The final survey comprised 20 questions and, while no personally identifying information was collected in the survey, respondents were asked to provide basic demographic data including gender, age group, post code, occupation, highest level of education attained, number of years spent working in that capacity and current area of work (primary care, community care, acute care etc.). Respondents were also asked to rate their level of self-confidence when caring for a person with dementia, the importance (to them) of having a high level of dementia education to enable them to do their job well and the extent to which the organisation for which they worked valued high quality care for people with dementia. These questions were answered using a 5-point Likert scale ranging from ‘Not at all’ to ‘Very/Extremely’. Respondents were also asked whether they had attended any dementia education or training activities within the past 12 months and to indicate what those activities were. Dementia education/training activities included a broad range of activities such as in-service training, dementia specific conferences, formal undergraduate or postgraduate courses, self-directed learning (reading books, watching DVDs or accessing online resources), online/multimedia courses provided through the workplace or attendance at a dementia lecture provided by Alzheimer’s Australia, a Dementia Training Study Centre (funded by the Australian government) or other recognised dementia education provider.

The 19-item Approaches to Dementia Questionnaire (ADQ) [4,5], that aims to capture respondent’s attitudes towards people with dementia, was also included in the survey. The reported test-retest reliability for the ADQ is good (Total=0.76, Hope=0.70, Person-Centeredness=0.69). Respondents rated the extent to which they agreed with 19 statements about dementia using a 5-point Likert scale ranging from ‘Strongly Disagree’ to ‘Strongly Agree’. A total score and two sub-scores ‘Hope’ and ‘Person-Centeredness (PC)’∥, can be derived and higher ADQ scores are indicative of more positive attitudes towards people with dementia; the Hope dimension reflects a sense of optimism/pessimism towards the abilities and the future of people with dementia while the PC dimension refers to the way in which people with dementia are recognised and responded to as unique individuals with the same value as any other person [6]. Possible scores for the ADQ Total scale range from 19 to 95; possible scores for the ADQ Hope subscale range from 8 to 40 and possible scores for the ADQ PC Scale range from 11 to 55.

### Ethics approval

Ethical approval for the project was obtained from both the Queensland University of Technology (QUT) and the Queensland Health (QH) Human Research Ethics Committees prior to survey distribution. A Consent Form was not required as completion of the survey was considered to constitute informed consent.

### Survey distribution

The target audience included registered nurses and allied health professionals working in Queensland, excluding Dentists, General Practitioners (GPs) and specialist medical practitioners. In the first instance, the survey was distributed via email to people listed on the Queensland Dementia Training Study Centre (Qld DTSC) and partner organisations’ databases (approximately 2,800 email addresses), that contained details of people with an expressed interest in dementia education/training. The survey was also distributed via email to nurses and allied health personnel working in QH facilities in six of the 16 Health Service districts into which Queensland is divided for the provision of adult health services. The number of surveys distributed to QH personnel is not known as the Directors of Nursing and Allied Health in each district distributed the survey to their staff on our behalf.

The survey was open for completion from June until October 2012.

### Data analysis

Demographic characteristics of respondents are presented as descriptive data only. Post codes were mapped to one of five geographical locations: Major Cities, Inner Regional, Outer Regional, Remote and Very Remote, defined using the Australian Standard Geographical Classification – Remoteness Area (ASGC-RA) [12]. Because there were very few respondents from ‘Very Remote’ Queensland, responses received from ‘Remote’ and ‘Very Remote’ Queensland were collapsed into one category - ‘Remote/Very Remote’. Differences between groups were assessed using the chi-squared statistic for categorical data.

Regression analyses were undertaken to investigate relationships between respondent characteristics and self-rated confidence in caring for people with dementia; and between respondent characteristics, self-rated confidence in caring for a person with dementia and responses to the ADQ. Variables entered into each model included all variables found to significantly predict the outcome in a bivariate analysis at p<0.05. Variables included in the model for self confidence in caring for a person with dementia were age group, occupation, length of time working in this capacity, current area of work, amount of time spent working with older people (aged 65 years and older), amount of time spent working with people with dementia and participation in dementia education or training in the past 12 months. Variables included in the analysis for ADQ Total and sub-scale scores were number of people supervised in the workplace, geographic location, self-confidence in caring for a person with dementia, participation in dementia education or training in the past 12 months and the importance of having a high level of dementia knowledge to enable you to do your job well. Dummy variable coding of categorical variables was undertaken prior to analysis and the most parsimonious model was retained as the final solution (see Tables 1 and 2). All analyses were completed using SPSS Version 19 [13].

**Table 1:** Important predictors of self-confidence when caring for a person with dementia

**Predictors**	**B**	**Standard error**	***β***	**p**
**Current area of work**				
Acute care (Reference category)				
Primary care	0.29	0.24	0.05	0.200
Community care	0.32	0.09	0.16	0.001
Residential care	0.46	0.10	0.21	0.000
Other	0.59	0.14	0.18	0.000
**Occupation**				
Registered Nurse (Reference category)				
Physiotherapist	−0.22	0.18	−0.05	0.226
Occupational Therapist	−0.44	0.13	−0.14	0.001
Speech Pathologist	−0.13	0.20	−0.03	0.505
Psychologist	−0.29	0.16	−0.08	0.064
Social Worker	−0.12	0.15	−0.03	0.404
Other	0.02	0.13	0.01	0.908
**More time working with people with dementia each week**	0.27	0.03	0.38	0.000
**More time working in this capacity**	0.07	0.03	0.90	0.026
**(Constant)**	2.57	0.17		

R^2^ = 0.28, Adj R^2^ = 0.26, p<0.001.

**Table 2:** Significant predictors of ‘Approaches to Dementia Questionnaire’ scores

**ADQ scale**	**Predictors**	**B**	**Standard error**	***β***	**p**
**ADQ total score**	Regarding having a high level of dementia knowledge as more (rather than less) important	1.70	0.45	0.19	0.000
	Greater self-confidence in caring for a person with dementia	1.38	0.39	0.17	0.000
	Participation in dementia education/training activity in the past 12 months	2.55	0.71	0.16	0.000
	(Constant)	64.93	1.78		
	R^2^ = 0.15, Adj R^2^ = 0.15, p<0.001				
**ADQ hope score**	Higher self-confidence in caring for a person with dementia	0.77	0.23	0.16	0.001
	Participation in dementia education/training activity in the past 12 months	1.51	0.42	0.16	0.000
	Regarding having a high level of dementia knowledge as more (rather than less) important	0.58	0.26	0.11	0.027
	(Constant)	23.29	1.05		
	R^2^ = 0.10, Adj R^2^ = 0.98, p<0.001				
ADQ Person-centeredness score	Regarding having a high level of dementia knowledge as more (rather than less) important	1.15	0.26	0.22	0.000
	Higher self-confidence in caring for a person with dementia	0.69	0.22	0.15	0.002
	Participation in dementia education/training activity in the past 12 months	0.99	0.40	0.11	0.014
	(Constant)	41.22	1.01		
	R^2^ = 0.14, Adj R^2^ = 0.13, p<0.001				

## Results

A total of 524 respondents completed the survey. It was not possible to determine a response rate as the survey was distributed widely through multiple avenues and the total number of survey recipients is not known. However, a majority of responses (n=445; 85%) were received from those who had received the survey via Qld DTSC and partner organisations’ email lists.

Key demographic characteristics of respondents are displayed in Table 3. Survey respondents were predominantly female (94%), although approximately a quarter of respondents did not provide this information (n=126; 24%). The majority (n=301; 59%) worked in major metropolitan areas while a very small minority worked in remote/ very remote (n=17; 3%) areas of Queensland. Most were registered nurses (60%), aged between 41 and 60 years (66%) and the majority worked in acute care (31%), community care (31%) and residential care settings (26%). Overall, respondents had considerable work experience with most reporting they had worked in their occupation for more than 10 years (57%), and around 40% reported working with older people with dementia at least half of the time. Respondents who worked in community and residential settings spent significantly more time working with people with dementia than those working in acute, primary and ‘other’ care settings (χ^2^ (16) = 126.8; p<0.001). A high level of confidence in caring for people with dementia was reported with approximately 70% respondents reported feeling ‘moderately’ (42%), or ‘very confident’ (27%) when caring for a person with dementia. Almost 90% of respondents reported that it was ‘very’ (43%) or ‘extremely important’ (43%) to have a high level of dementia knowledge to enable them to do their job well and a high level of recent participation in dementia education activities was reported by respondents with almost two-thirds (67%) indicating they had participated in some form of dementia education or training activity within the past 12 months. Respondents who reported that a high level of dementia knowledge was ‘very’ or extremely’ important to enable them to do their job well were significantly more likely to have undertaken some dementia education or training activity in the past 12 months than others (χ^2^(4) =45.4; p<0.001). In-service training (n=231; 44%), self-directed learning (n=228; 44%) and attendance at an event organized by the Qld DTSC (n=222; 42%) were the activities respondents most frequently reported participating in over the past 12 months.

**Table 3:** Demographic characteristics of survey respondents

**Characteristic/Response**	**n (%)**
**Gender**	Female = 375 (94.2)
(Total n = 398)	Male = 23 (5.8)
**Age group**	
21–40 years	123 (23.6)
41–60 years	330 (65.7)
61 years and older	53 (10.7)
(Total n = 524)	
**Occupation**	
Registered Nurse	316 (60.3)
Physiotherapist	21 (4.0)
Occupational Therapist	48 (9.2)
Speech Pathologist	18 (3.4)
Psychologist	32 (6.1)
Social Worker	37 (7.1)
Other^#^	51 (9.9)
(Total n = 524)	
**Geographic location**	
Major city	301 (59.6)
Inner regional	128 (25.3)
Outer regional	59 (11.7)
Remote/Very remote	17 (3.4)
(Total n = 505)	
**Length of time working in this role**	
Less than 12 months	25 (4.8)
1–2 years	43 (8.3)
3 – 5 years	92 (17.7)
6–10 years	63 (12.1)
More than 10 years	297 (57.1)
(Total n = 520)	
**Current area of work**	
Acute care^§^	161 (30.8)
Primary care	16 (3.1)
Community Care	163 (31.2)
Residential Care	136 (26.0)
Other^##^	47 (9.0)
(Total n = 523)	
**Highest level of education attained**	
Bachelor’s degree/Certificate in Nursing	319 (61.1)
Graduate Certificate/Graduate Diploma	65 (12.5)
Master’s degree/PhD/Professional doctorate	98 (18.8)
Other^¶^	40 (7.7)
(Total n = 522)	
**How much time at work is spent working with people with dementia?**	
Hardly any time (0-9% of my time)	88 (16.9)
A little time (10-24% of my time)	97 (18.5)
Some of the time (25-49% of my time)	125 (24.0)
A moderate amount (50-74% of my time)	115 (22.1)
Most of my time (>75% of my time)	95 (18.1)
(Total n = 520)	
**How important is it for you to have a high level of dementia knowledge to enable you to do your job well?**	
Not at all important	5 (1.0)
A little important	17 (3.3)
Moderately important	46 (8.8)
Very important	227 (43.3)
Extremely important	223 (42.6)
(Total n = 518)	
**How confident do you feel when caring for a person with dementia?**	
Not at all confident	5 (1.0)
A little confident	42 (8.1)
Somewhat confident	111 (21.2)
Moderately confident	220 (42.0)
Very confident	141 (27.2)
(Total n = 519)	
**Participation in dementia education/training in the last 12 months**	347 (67.0)

^#^Other occupation includes podiatrists, dieticians/nutritionists and others.

^§^Acute care was deemed to include sub-acute, outpatient and emergency services.

^##^Other area of work primarily included staff working in education and research.

^**¶**^Other includes Other Diplomas/Certificates, Associate degrees and other postgraduate studies.

### Self-confidence in caring for a person with dementia

Results of the regression analysis showed that four variables significantly contributed (F=15.90, p <0.001) to higher self-confidence in caring for a person with dementia. Together, the four variables (listed in Table 1) accounted for 25.8% of the variance in self-confidence. Compared to those working in acute care settings, healthcare professionals working in residential care, community care and ‘other’ settings had significantly higher confidence levels. Registered nurses displayed the highest confidence levels, but only the difference with occupation therapists (the next largest group numerically), was statistically significant. Greater confidence was also evident in those who spent more time working with people with dementia and in those with more work experience.

### Attitudes towards dementia

The overall average ADQ Total score was 79.06 (SD = 7.54), the average Hope score was 29.70 (SD = 4.36) and the average PC score was 49.36 (SD = 4.27). These scores indicate generally positive attitudes, being close to the maximum possible for each scale. Results of the regression analysis indicated that three variables: considering that having a high level of dementia knowledge to enable one to perform one’s job well as more, rather than less important, higher levels of self-confidence in caring for a person with dementia and participation in dementia education or training in the past 12 months, were significantly predictive of higher ADQ Total scores (F=27.46, p< 0.001), together accounting for 14.5% of the variance in ADQ Total scores. The same three variables were also significantly predictive of higher ADQ Hope scores (F=18.68, p< 0.001), together accounting for 9.8% of the variance; and higher ADQ personhood scores (F=25.43; p<0.001), accounting for 13.3% of the variance (see Table 2).

## Discussion

Unsurprisingly most respondents were registered female nurses, aged between 41–60 years of age (65.6%) , which is consistent with the average age of employed nurses in Australia [14]. Overall, respondents had considerable experience in working with people with dementia, high levels of self-confidence in caring for this population and positive attitudes towards people with dementia. A high level of dementia knowledge was regarded as important by most respondents which is borne out by the high level (around two-thirds of respondents) of participation in dementia education or training in the past 12 months. A majority of survey responses were received from healthcare professionals whose emails were registered with the Qld DTSC and partner organisations. People register on these websites to receive information regarding dementia education and training opportunities and hence, it is likely that survey respondents may have a higher than average level of experience of working with people with dementia and greater than average self-confidence in caring for people with dementia. As a consequence, the survey findings may not be generalizable to the entire healthcare workforce, particularly those working in primary care settings, nor to males who were under-represented in our sample. Nevertheless, important associations between experience in providing dementia care, self-confidence in caring for people with dementia, positive attitudes towards people with dementia and recent participation in dementia education or training activities can be identified from the survey results. Overall, the results support our initial hypothesis that there would be positive relationships between education, training and self-confidence and positive attitudes towards people with dementia.

Important predictors of self-confidence in this study were being a registered nurse (although the difference was significant only in comparison to occupational therapists) working in a community care, residential care or ‘other’ setting (primarily education and research settings) compared to an acute care setting, working for more years in one’s role and spending more hours per week working with people with dementia. Thus, as might be expected, self-confidence increases with more experience caring for people with dementia. A similar finding was reported by Hughes and colleagues [9] who reported that being a senior staff carer was associated with higher self-confidence in caring for people with dementia. They also reported that having more dementia knowledge and having undertaken dementia specific training also explained higher self-confidence scores. While participation in dementia education or training in the past 12 months did not emerge as a significant predictor of self-confidence in our study, it is likely that knowledge of dementia care increases the longer a person works in their role and spends more time working with people with dementia. This may account for our finding that respondents who worked in community and residential care settings were more confident in caring for people with dementia as respondents who worked in those settings spent significantly more time working with people with dementia than those working in acute or primary care settings (of whom there were very few in this study).

Greater self-confidence, in turn, was predictive of more positive attitudes towards people with dementia. Other important factors predictive of more positive attitudes were participation in dementia education or training in the last 12 months and the belief that such education was more (rather than less) important to enable people to perform their job well. While those who considered such education to be very important were more likely to have participated in dementia education or training in the last 12 months, it may also be that those who value dementia education most highly are those who are able to appreciate the role of dementia education in providing good quality care. The results of this study, however, do not allow this question to be conclusively answered and further research is required to better understand why this belief is important in predicting positive attitudes towards people with dementia.

Our findings are consistent with previous reports that care staff who have a good knowledge of dementia care also have more positive attitudes towards people with dementia [4,5,8], and with those of Kada [15] who reported that staff with specialised training in dementia care had significantly higher hope attitudes compared to staff who had not undertaken such training. More positive attitudes towards people with dementia, in turn, have been shown to be related to positive care staff behaviours for the person with dementia, such as positive engagement and stimulation, and better self-reported quality of life in people with dementia living in aged care facilities [4-7,16,17]. Staff training in dementia has also been shown to result in improvements in practice [18], and better quality of life in residents of long-term care facilities with dementia [6,7].

Hence, our findings underscore the importance of regular participation in dementia education/training to foster a competent, confident healthcare workforce and indicate that regular participation in dementia education and training should be strongly endorsed. They reinforce the views expressed earlier by members of our research team [11], that systematic dementia-specific education or training should be introduced for health care workers to improve healthcare delivery for people with dementia. Our findings also indicate that it is important to retain older, experienced healthcare staff in the workforce who are likely to play an important role in mentoring less experienced staff.

### Limitations

In addition to the issue of generalizability discussed earlier, an additional limitation of the present study, due to the requirement that the survey be brief, was the absence of objective measures of dementia knowledge and competence in providing care for people with dementia. Hence, the level of dementia knowledge and competence in providing care for people with dementia of these survey respondents is not known and while it has previously been reported that greater self-confidence in caring for people with dementia is associated with better care practices for people with dementia, no such conclusion can be drawn from the present study. While survey respondents appear to represent an experienced, dedicated workforce keen to improve their knowledge of dementia care, the extent to which this translates into knowledge and competence in providing care for this population was not demonstrated in this study.

## Conclusions

Participation in dementia education/training and self-confidence in caring for people with dementia are important predictors of more positive attitudes towards people with dementia. Because more positive attitudes have been shown by others to be related to improved practice and better quality of life for people with dementia living in residential aged care facilities, it is recommended that regular dementia education/training be provided and promoted for all healthcare personnel who work with people with dementia. In particular, systematic up-skilling of staff working in acute care, because they report lower confidence yet care for a high proportion of the most acutely unwell people with dementia, including those who are dying, needs to be made a priority.

## Abbreviations

ADQ: Approaches to dementia questionnaire; ADQ PC: Approaches to dementia questionnaire person-centeredness subscale; ADQ Hope: Approaches to dementia questionnaire hope subscale; Qld DTSC: Queensland Dementia Training Study Centre.

## Competing interests

The authors declare that they have no competing interest.

## Authors’ contributions

EB and M-K were primarily responsible for developing the study concept and design, and obtaining financial support for the project. CT was responsible for overseeing the data collection, data analysis and drafted the manuscript. EF provided statistical advice. All authors read and approved the final manuscript.

## Pre-publication history

The pre-publication history for this paper can be accessed here:

http://www.biomedcentral.com/1471-2318/13/101/prepub
